# Structure of Relationships Between the University Organizational Image and Student Loyalty

**DOI:** 10.3389/fpsyg.2021.727961

**Published:** 2021-07-29

**Authors:** José Álvarez-García, María de la Cruz del Río-Rama, Cristiana Oliveira, Amador Durán-Sánchez

**Affiliations:** ^1^Financial Economy and Accounting Department, Faculty of Business, Finance and Tourism, University of Extremadura, Cáceres, Spain; ^2^Business Management and Marketing Department, Faculty of Business Sciences and Tourism, University of Vigo, Ourense, Spain; ^3^European University of the Canary Islands, Santa Cruz de Tenerife, Spain

**Keywords:** organizational image, university, loyalty, satisfaction, S-U identification, higher education

## Abstract

The aim of this research is to contrast an explanatory model of how the perceived organizational image of a university center (faculty) influences its students' loyalty. The data is obtained from a structured survey of students in Spain, obtaining a sample of 224 valid questionnaires. The methodology used is exploratory and confirmatory factor analysis to validate the measurement scales and the estimation of the model is carried out by applying Structural Equation Modeling (SEM). The results show that organizational image is the key variable to influence students' decision to continue taking new courses at the center, as well as to recommend it to other people. It is observed that the greater students' positive perception of the organizational image is, the greater their satisfaction with the center will be, which results in a higher level of loyalty to the center in which they study. However, their identification levels with the center is not a relevant variable in the process of increasing loyalty.

## Introduction

At present, there are several factors regarding different aspects that significantly increase competition among university centers in Spain, as well as in many other countries; attracting new students, attracting financial resources, etc. Among these factors is the decreasing birth rate that leads universities to have to compete for students, the globalization of the economy (Altbach, 2004), the economic crisis of the last decade, European convergence and the Declaration of Bologna, free movement of students and the emergence of private centers in educational markets. Therefore, in this context, the organizational image/University or Faculty image is established as one of the main intangible assets of an educational institution (Šontaite and Bakanauskas, 2011; Stergiou and Tsikliras, 2014; Collins and Park, 2016; Plewa et al., 2016).

In fact, there are many benefits associated with organizational image (Pérez and Torres, 2017: 128): it improves the functioning and competitiveness of the university organization (Treadwell and Harrison, 1994; McPherson and Schapiro, 1998; Druteikiene, 2011; Blázquez and Peretti, 2012), it contributes to achieving student loyalty and improving their satisfaction (Helgesen and Nesset, 2007; Stevens et al., 2008; Polat, 2011), it attracts and retains human resources and produces a positive response in workers (Treadwell and Harrison, 1994; Nolan and Harold, 2010).

Taking into account the benefits produced by organizational image, it becomes a very important intangible asset to attract and retain/loyalty from the best students (Helgesen and Nesset, 2007; Stevens et al., 2008; Polat, 2011). Therefore, it is essential to establish, manage and maintain an image that enables to create a competitive advantage and to differentiate itself from the competition in current markets (Paramewaran and Glowacka, 1995).

There is a wide variety of studies focused on the University image from different perspectives (Arpan et al., 2003; Magierski and Kassouf, 2003; Kazoleas et al., 2011; Pérez and Torres, 2017). However, there are fewer that study the relationship of this concept with others, such as student satisfaction and loyalty (Beerli et al., 2002; Beerli and Díaz, 2003; Helgesen and Nesset, 2007; Brown and Mazzarol, 2009; Cervera et al., 2012; Chandra et al., 2019; Hassan and Shamsudin, 2019). For this reason, a greater research effort is required regarding this concept and in particular how this concept influences student loyalty. According to Cervera et al. (2012: 8), “the University image constitutes an emerging research topic…, since academic research on this topic has traditionally been focused on the study of the issuer…, overlooking those approaches whose object of study was focused on the receptor.”

Following this line of work, the aim of this research is to contrast an explanatory model of how the organizational image perceived by the students of a university center influences their loyalty to the center, as well as their Student-University (S-U) identification. In order to achieve this objective and corroborate the proposed working hypotheses, the data was collected from a structured questionnaire that was completed by 224 students from a University Center in Spain. The Structural Equation Modeling technique is applied to contrast the proposed hypotheses.

The novelty of this study lies in analyzing the structure of relationships between the organizational image as a multidimensional construct (orientation/training, reputation, aesthetic/affective) in loyalty, considering satisfaction and S-U identification as intermediate constructs. Understanding this structure will allow university center managers to know which dimension/s of the organizational image to focus their efforts on in order to improve their students' satisfaction and consequently increase their loyalty toward the center.

The paper is structured as follows. After this introduction, the theoretical framework that supports the research is introduced and the working hypotheses are presented in Literature Review and Hypotheses section. The methodology is described in Methodology section; sample, questionnaire and data analysis. The empirical results are shown below. Finally, the results are discussed, the main conclusions are drawn and the limitations of the research are discussed.

## Literature Review and Hypotheses

### Organizational Image

One of the first definitions of organizational image was proposed by Kotler (1975), with both a beliefs and attitudes approach regarding an organization; the sum of beliefs, ideas and impressions that a person has on an object (Kotler and Fox, 1995). Beerli et al. (2002) subsequently state that it is a rational and emotional assessment and interpretation that a person makes of the organization, in which two components are integrated: cognitive (beliefs) and affective (emotions and feelings) components. Along the same lines, Kazoleas et al. (2011) states that the image is generated in subjects. Therefore, there can be as many images as there are subjects and they consider that this is the result of the interpretation made by the subjects regarding the information or disinformation provided. Following this trend, Polat (2011) defines it as the vision, representation or impression that people form in their minds based on the information or data of an organization, obtained from the interaction with the elements or components of the organization. In the university context, the university organizational image was defined by Nguyen and Leblanc (2001) as the perceived image that its students have according to their ideas, interests and personal, social and background experiences. Students make a rational and emotional assessment of the tangible and intangible attributes of the organization.

Taking into account the large number of approaches to define organizational image, it is revealed that it is a complex and multidimensional concept (Nguyen and Le Blanc, 2002; Arpan et al., 2003; Beerli and Díaz, 2003), as it is based on the perception and differentiating and comparative assessment of the characteristics of an organization, which is carried out by a person (Günalan and Ceylan, 2014). In this regard, the image will be influenced by the beliefs, stereotypes, ideas, significant behaviors and impressions that a person has of an organization (Kotler and Andreasen, 2008). Specifically, Arpan et al. (2003) mentioned three factors: academic factors, athletic factors and the extent of news coverage of the university.

Regarding the organizational image components, there is currently no consensus. This research follows the approach of Beerli et al. (2002) and the following are considered cognitive and affective components: university orientation and training, reputation, overcrowding, accessibility, age, affective.

In this context, the following hypothesis is proposed:

H1: The perceived image of the Faculty is a multidimensional construct with cognitive and affective components.

### Consequences of the Perceived Organizational Image

#### Relationship Between Organizational Image and Loyalty

Jacoby and Kyner (1973) propose that loyalty can be defined by taking into account two perspectives, on the one hand, the attitudinal perspective and on the other hand, the behavioral perspective. Thus, Dick and Basu (1994) define customer loyalty as the relationship between the relative attitude and repetitive purchase pattern. In this regard, many investigations carried out consider this dual perspective in very different contexts (Söderlund, 1998; Homburg and Giering, 2001; Rodríguez et al., 2020; López-Sanz et al., 2021). The important role of loyalty for the survival of educational institutions is evident (Helgesen and Nesset, 2007). In this regard, if a student with loyalty is achieved, he/she can attract others by positive word-of-mouth communication and maintain a lasting relationship with the university institution. In the university education context, there are studies that corroborate the relationship between University image and loyalty (Martensen et al., 1999; Nguyen and Leblanc, 2001; Beerli et al., 2002; Beerli and Díaz, 2003; Alves and Raposo, 2010). Therefore, the following hypothesis can be proposed:

H2: The perceived image of the Faculty influences loyalty toward it directly and positively.

#### Relationship Between Organizational Image and Satisfaction

Satisfaction is a complex concept that depends on the context of analysis where it is involved (Giese and Cote, 2000), therefore, there are many definitions. In this research context, the definition pecifically proposed in the educational field by Elliot and Healy (2001) is taken into account “it is a short-term attitude that results from assessing their experience with the educational service received”. Thus, a comparison between expectations and results is produced (Oliver, 1980; Anderson, 1994) and all aspects that make up a relationship are evaluated, including image (Sanzo et al., 2003). This relationship is empirically supported in the literature in the University image context (Martensen et al., 1999; Nguyen and Leblanc, 2001; Beerli et al., 2002; Helgesen and Nesset, 2007; Alves and Raposo, 2010). Thus, the following hypothesis is proposed:

H3: The image perceived by the students of the Faculty influences their satisfaction with the service received directly and positively.

#### Relationship Between Organizational Image and S-U (Student-University) Identification

The concept of S-U identification is investigated from different areas of knowledge following a personal or social perspective (Fernandes et al., 2009). In this research, we are interested in the study of the relationship between people (students) and organization (University). According to Tesser et al. (1988), students will be attracted to a university when they perceive that it has characteristics similar to their own and with which they are capable of sharing feelings, opinions or values. It refers to the student's psychological attachment to his University.

This concept is defined by Ashforth and Mael (1989) as the recognition, manifestation of affinities and the attraction that comes from a process of internalization and incorporation of beliefs, values and attitudes of a social group. Subsequently, they define it as “one's perception of belonging to an organization, where the person is defined in terms of the organization of which he/she is a member” (Mael and Ashforth, 1992: 104). Bhattacharya and Sen (2003) define it as the cognitive state of connection and proximity of a consumer (student) with an organization. According to these authors, this state is generated as a result of a process of comparison between personal identity and that of the organization carried out by the consumer (student). If the student perceives that he/she shares the same traits, values and attributes with the organization, he begins to define his own self-concept based on his relationship with the organization.

Several studies analyse the relationship between the perceived image of the organization or some of its dimensions with SU identification (Dutton et al., 1994; Bhattacharya et al., 1995; Kreiner and Ashforth, 2004; Ahearne et al., 2005; Cornwell and Coote, 2005). For all these reasons, the following hypotheses can be proposed:

H4: The image perceived by the students of the Faculty influences their identification with it directly and positively.

On the other hand, Marín and Ruiz (2007) confirm that this cognitive state will influence the affective states of the consumer regarding the organization, and as a result, their loyalty. As a consequence of the above, universities want to create and maintain this identification as strong as possible as a means of strengthening loyalty to the university.

H7: S-U identification of students with the Faculty influences their loyalty toward it directly and positively.

#### Relationship of Satisfaction With Loyalty and S-U Identification

(Dermanov and Eklöf, 2001) state that obtaining customer satisfaction involves certain consequences that condition their future activity, a student who is satisfied with the service received may develop various attitudes and behaviors that are indicative of loyalty (Marzo et al., 2005). In this regard, students who are satisfied with the service can show their intention to return (Patterson et al., 1997; Athiayaman, 2000; Lervik and Johnson, 2003) and will certainly recommend it to others (Mavondo and Zaman, 2000; Tsarenko and Mavondo, 2001). Many authors corroborate that satisfaction is an antecedent to student loyalty (Alves and Raposo, 2004; Gonçalves et al., 2004; Schertzer and Schertzer, 2004; Taylor et al., 2004; Tsarenko et al., 2004; Marzo et al., 2005). Thus, the following hypotheses are proposed:

H5: The satisfaction perceived by the students influences their loyalty to the Faculty directly and positively.
H6: The satisfaction perceived by the students influences their S-U Identification with the Faculty directly and positively.

**Figure 1:**
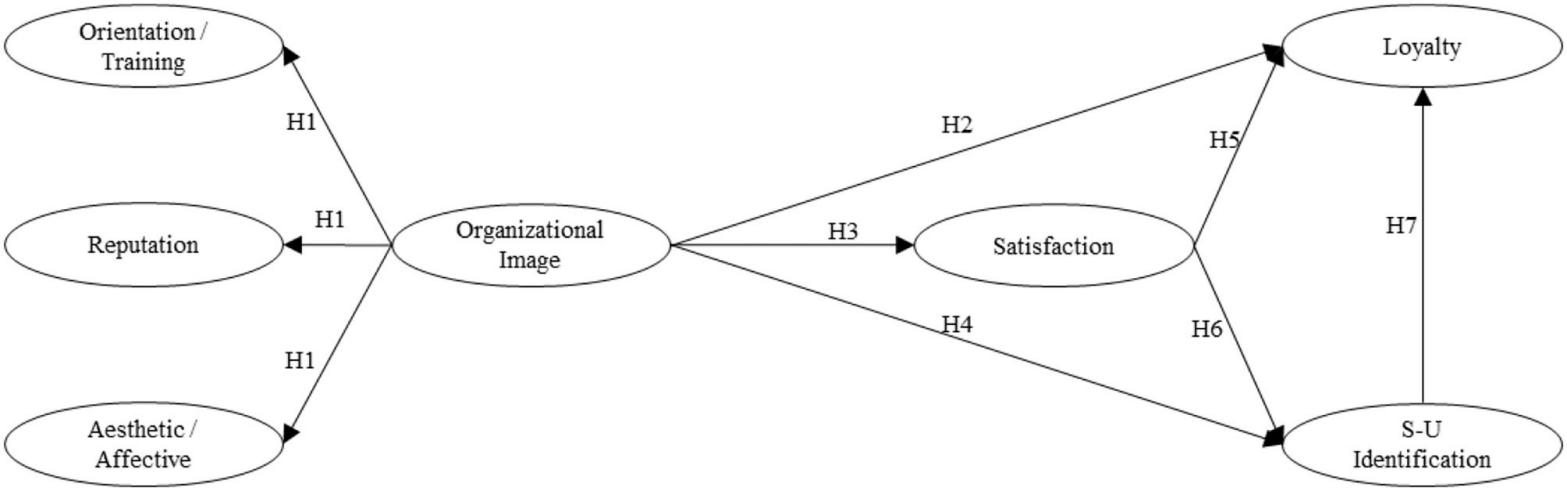
Theoretical model and hypothesis. Source: Authors' own data.

To summarize, in Figure 1 the “path diagram” is shown.

## Methodology

### Universe Study, Questionnaire and Measurement

The target population is 1,486 students enrolled at a University Center in Spain and who are studying Finance and Accounting, Business Administration, Tourism and the Double Degree in Business Administration and Tourism.

A structured questionnaire was designed to measure the constructs included in the model. Some of the most relevant measurement scales in the literature were taken as reference and adapted to the specific characteristics of the target population. The use of items used in other investigations enables to fulfill the internal validity requirement. Organizational image (25 items) is a scale adapted from Cervera et al. (2012) and Pérez and Torres (2017), which are an adaptation from the Beerli and Díaz (2003) scale and Beerli et al. (2002). Satisfaction (3 items), which is a scale of Schlesinger et al. (2014), adapted from Fornell (1992). Loyalty (4 items) is a scale adapted from Cervera et al. (2012), which is an adaptation from the scale of Martensen et al. (1999) and Hennig et al. (2001) and Student-Faculty Identification (S-U) (6 items), which is a scale adapted from Cervera et al. (2012) and is an adaptation from the scale of Mael and Ashforth (1992) and Bhattacharya et al. (1995). A 5-point Likert scale was used, with 1 being “totally disagree” and 5 “totally agree”.

The data collection process was carried out on-line during May through the GoogleForms platform. A sample of 224 valid questionnaires (response rate of 15.07%) was obtained, which implies a sampling error of 6% for a confidence level of 95%, Z = 1.96, *p* = *q* = 0.50. Regarding the profile of the respondent, of the 224 respondents, 144 belong to the female gender and 80 to the male, and are part of an age range that goes from 18 years to 43, with the average age being 22.44 years (standard deviation of 3.75). As the data are cross-sectional, obtained from a single source, the Common Method Bias (CMB) was verified by applying The Harman single-factor test (Podsakoff and Organ, 1986); the non-existence of the common method problem in this research is confirmed.

### Data Analysis

To perform the descriptive and exploratory factor analysis of the data, the statistical programme SPSS 19.0 (Statistical Package for the Social Sciences) and AMOS 20.0 (Analysis of Moment Structures) software were used for confirmatory factor analysis of the scales and estimation of the causal model proposed.

#### Validation of the Measurement Model

Following, Anderson and Gerbing (1988), the psychometric properties of the scales were analyzed. Firstly, the reliability of the scales is analyzed; the item-total correlation should take values> 0.3 (Nurosis, 1993) and Cronbach's alpha> 0.7 (Nunnally, 1979). Regarding the Unidimensionality that allows to identify the dimensions of the scales, it was validated in two stages: (1) Exploratory Factor Analysis (EFA) of principal components with varimax rotation (Bagozzi and Baumgartner, 1994), (2) Confirmatory Factor Analysis (CFA), which allows to examine the measurement model (reliability and validity of measures), the structural model and the global model of each of the scales.

The following are considered minimum levels: (1) the loadings> 0.05 (Hair et al., 1999) and the percentage of the explained variance> 50%, (2) to examine the fit of the structural measurement model, the critical Ratio for regression weight must exceed ±1.96 and the standard regression weight (β)> 0.5 (Jöreskog and Sörbom, 1993), (3) to examine the global model, the goodness-of-fit indices of the model are observed (Joreskog and Sörbom, 1988; Mangin and Mallou, 2006): values above 0.9 are recommended (Hair et al., 1999) for the comparative fit index (CFI), goodness of fit index (GFI), normed fit index (NFI), adjusted goodness of fit index (AGFI). The robustness of mean squared error approximation index (RMSEA), according to Steiger (1990), Browne and Cudeck (1993), should be < 0.08 and values between 2 and 3 are recommended (Jöreskog and Sörbom, 1993) for Normalized χ^2^ (χ^2^/df). To finish, the reliability is estimated again through composite reliability (CR) > 0.7 and extracted variance (AVE)> 0.5.

#### Estimation of Structural Equation Modeling (SEM)

The Structural Equation Modeling (SEM) technique or covariance structure model is used to test the causal relationships proposed in the theoretical model. To estimate the parameters, the Method of Maximum Likelihood (ML) was used and the bootstrap technique (5,000 samples) was applied taking into account the absence of normality. The methodology proposed by Mangin and Mallou (2006) is followed: parameter estimation, adjustment evaluation, re-specification of the model and interpretation of results. To evaluate the structural and global model, the indices already mentioned in the validation of the scale and (β) standard regression weight (critical coefficient> ±1.96) are considered. *R*^2^ measures the variance of the construct that is explained by the model.

## Results

### Measurement Model

The analysis applied to check the reliability of the scales indicates an adequate internal consistency of the scales; correlation-total item> 0.3, not being necessary to eliminate any item and Cronbach's alpha is higher than the recommended minimum of 0.7. An EFA was applied (maximum likelihood extraction method and Varimax rotation) to check for unidimensionality. All measurement scales are unidimensional except for the organizational image scale, which has three factors that explain 57.43%> 50% and Loadings> 0.5 (recommended minimums) (Table 1).

**Table 1:** Descriptive findings and exploratory factor analysis (reliability and validity of scales).

**Factors**		**Scale items^**a**^**	**Mean**	**(s.d.)^**b**^**	**Exploratory factor analysis (loadings)**			
					**Factor 1**	**Factor 2**	**Factor 3**	**Bartlett's test of Sphericity Kaiser-Meyer_Oklin index^**c**^**
Organizational Image Scale	Orientation/training (α Cronbach: 0.909)	IM1	3.19	0.99	0.685			χ^2^(sig.): 4077.787 (0.000)
		IM2	2.90	1.08	0.761			KMO: 0.923
		IM4	2.94	1.09	0.662			Measure of simple adequacy: (0.878–0.875)
		IM5	2.93	1.01	0.666			
		IM6	2.50	1.10	0.568			% Variance: 57.432
		IM9	3.00	0.94	0.714			
		IM10	2.69	1.09	0.567			
		IM11	2.87	0.98	0.424			
		IM16	2.99	1.11	0.416			
		IM19	2.47	1.03	0.557			
		IM20	2.50	1.06	0.510			
		IM21	2.94	0.98	0.426			
	Reputation (α Cronbach: 0.914)	IM7	2.28	0.95		0.558		
		IM8	2.75	1.01		0.518		
		IM12	2.14	0.96		0.699		
		IM13	2.33	0.99		0.673		
		IM14	1.75	0.95		0.851		
		IM15	2.04	1.08		0.754		
		IM17	1.70	0.93		0.782		
		IM18	1.89	1.02		0.776		
	Aesthetic/affective (α Cronbach: 0.873)	IM3	3.49	0.92			0.569	
		IM22	3.36	0.91			0.830	
		IM23	2.55	0.94			0.713	
		IM24	2.64	1.00			0.690	
		IM25	2.96	1.06			0.845	
	Own value				6.033	5.525	3.949	
	% variance factor explained				22.345	20.461	14.626	
	% cumulative explained variance				22.345	42.806	57.432	
Satisfaction (α Cronbach: 0.914)		S1	3.19	1.14	0.933			χ^2^(sig.): 478.371 (0.000)
		S2	3.00	1.06	0.902			KMO: 0.747
		S3	3.15	1.15	0.937			Measure of simple adequacy: (0.721–0.710)
								% Variance: 85.405
Loyalty (α Cronbach: 0.914)		L1	2.44	1.17	0.859			χ^2^(sig.): 640.756 (0.000)
		L2	2.80	1.14	0.921			KMO: 0.840
		L3	3.09	1.12	0.860			Measure of simple adequacy: (0.879–0.806)
		L4	2.85	1.14	0.925			% Variance: 79.537
S-U Identification (α Cronbach: 0.876)		SU1	2.22	1.13	0.771			χ^2^(sig.): 689.363 (0.000)
		SU2	2.93	1.23	0.718			KMO: 0.859
		SU3	2.54	1.20	0.681			Measure of simple adequacy: (0.871-0.899)
		SU4	2.59	1.18	0.859			
		SU5	2.51	1.15	0.884			% Variance: 62.433
		SU6	2.90	1.24	0.808			

a *The items listed in this table have been summarized for ease of presentation and comprehension;*

b *b^s.d^: Standard deviation*.

c *Tests that show that the data obtained through the questionnaire are adequate to perform the factor analysis [requirements: Bartlett's Sphericity Test χ^2^ (sig. < 0.5), KMO > 0.7 median, > 0.8 y >0.9 muy bueno, MSA = unacceptable for values below 0.5]*.

*Source: Authors' own data*.

The analysis of unidimensionality is continued since the EFA is exploratory and the CFA is applied. In the specific case of the organizational image scale (multidimensional in the EFA), following, Hair et al. (1999), a strategy of rival models is developed (Table 2). Model 2 (oblique) of 1st order has a better adjustment than model 1 and 2 (orthogonal). It is then re-specified to improve the adjustment (Model 3) and compared to a 2nd order model (Model 4). The results confirm the multidimensionality of the scale, the optimal measurement model is model 4 of 2nd order.

**Table 2:** Fit índices for image scale.

**Models**	**χ^2^**	**df**	**χ^2^ (df)**	**P**	**GFI**	**AGFI**	**PGFI**	**TLI**	**CFI**	**RMSEA**
Model 1 (1 variable−25 items)	1356.094	275	4.931	0.000	0.630	0.562	0.533	0.674	0.701	0.133
Model 2−1st order (3 variables−25 items) (orthogonal)	1250.349	275	4.547	0.000	0.686	0.629	0.581	0.706	0.730	0.126
Model 2−1st order (3 variables−25 items) (Oblique)	945.028	272	3.474	0.000	0.724	0.670	0.606	0.795	0.814	0.105
Model 3 (model 2 re-specified−3 variables-21 items)	425.850	179	2.379	0.000	0.843	0.798	0.653	0.908	0.922	0.079
Model 4−2nd order (model 2 re-specified−3 variables-21 items)	412.373	178	2.317	0.000	0.849	0.804	0.654	0.912	0.926	0.077

*Source: Authors' own data*.

Table 3 shows the AFC results of the scales. Items IM1, 3, 16 and 21 are eliminated as they do not have significant factor loadings. The rest of the items have β > 0.50 and are significant (critical coefficient> ±1.96). The models have good measures of absolute, incremental and parsimony adjustment as all the indicators are within the generally accepted limits.

**Table 3:** Reliability and confirmatory factor analysis.

**Scales**		**β**	**CR**	**AV**	**Confirmatory factory analysis/Composite reliability test**	**Scales**	**β**	**CR**	**AV**	**Confirmatory factory analysis/Composite reliability test**
**Organizational image**	Orientation/ training		0.89	0.48	χ^2^(df5) = 412.373 (*p* = 0.000), GFI = 0.849, AGFI = 0.804, CFI = 0.926 RMSEA = 0.077 χ^2^Normalized(χ^2^/df) = 2.317	*Satisfation*		0.93	0.81	χ^2^(df5) = 100.650 (*p* = 0.001), GFI = 0.940, AGFI = 0.910, CFI = 0.981, RMSEA = 0.054 χ^2^Normalized(χ^2^/df) = 1.650
	IM2	0.672				S1	0.901			
	IM4	0.744				S2	0.824			
	IM5	0.743				S3	0.927			
	IM6	0.71								
	IM9	0.755								
	IM10	0.669								
	IM11	0.652								
	IM19	0.777								
	IM20	0.654								
	Reputation		0.91	0.57		*S-U*		0.86	0.51	
	IM7	0.803				*Identification*				
	IM8	0.635				SU1	0.751			
	IM12	0.799				SU2	0.606			
	IM13	0.797				SU3	0.602			
	IM14	0.797				SU4	0.889			
	IM15	0.738				SU5	0.878			
	IM17	0.691				SU6	0.731			
	IM18	0.723								
	Aesthetic/ affective		0.86	0.62		*Loyalty*		0.93	0.76	
	IM22	0.648				L1	0.794			
	IM23	0.888				L2	0.906			
	IM24	0.861				L3	0.809			
	IM25	0.69				L4	0.906			

*β, standard regression weight; CR, composite reliability; AV, average variance.*

*p < 0.001.*

*Source: Authors' own data*.

Reliability is measured through average variance (AV) that must be >0.5 and composite reliability (CR) > 0.7 (Table 3) (Bagozzi and Yi, 1988; Hair et al., 1999). The content validity was ensured by the literature review carried out, as well as by the pre-test performed. It is concluded that there is convergent validity since β > 0.5 and statistically significant (t-student> ±1.96) and AVE> 0.5.

Discriminant validity is confirmed. It was analyzed by examining three indicators: (1) confirmed if Cronbach's alpha of each scale is higher than any of the correlations between that scale and the rest, which was proved and (2) whether inter-factor correlations are less than the square root of the average variance extracted (Fornell and Larcker, 1981, Chin, 1988), (3) none of the confidence intervals contains the unit (Bagozzi and Yi, 1988). Taking into account the results, discriminant validity is confirmed (Table 4).

**Table 4:** Correlation matrix and discriminant validity.

	**Square root AV**	**(1)**	**(2)**	**(3)**	**(4)**	**(5)**	**(6)**
Orientation/training (1)	0.69	**0.909** ^**a**^	0.752^b^	0.605	0.598	0.650	0.329
Reputation (2)	0.75		**0.914**	0.554	0.494	0.585	0.303
Aesthetic/affective (3)	0.78			**0.873**	0.517	0.534	0.348
Satisfaction (4)	0.9				**0.914**	0.800	0.350
						0.114^c^ (0.198–0.478)	0.698 (0.202–0.634)
S-U identification (5)	0.71					**0.914**	0.416
							0.127
							(0.221–0.493)
Loyalty (6)	0.87						**0.876**

a * Shown in bold on the main diagonal are the Cronbach's alpha for each scale, which should be higher than the correlation between that scale and the rest*.

b *Inter-scale correlation.*

c *The squared correlation between pairs of factors (less than AVE) and confidence interval for the estimated correlations, ±twice the standard error, does not include the value of 1.*

*All significant at p < 0.01*.

*Source: Authors' own data*.

### Analysis of the Structural Models

**Figure 2:**
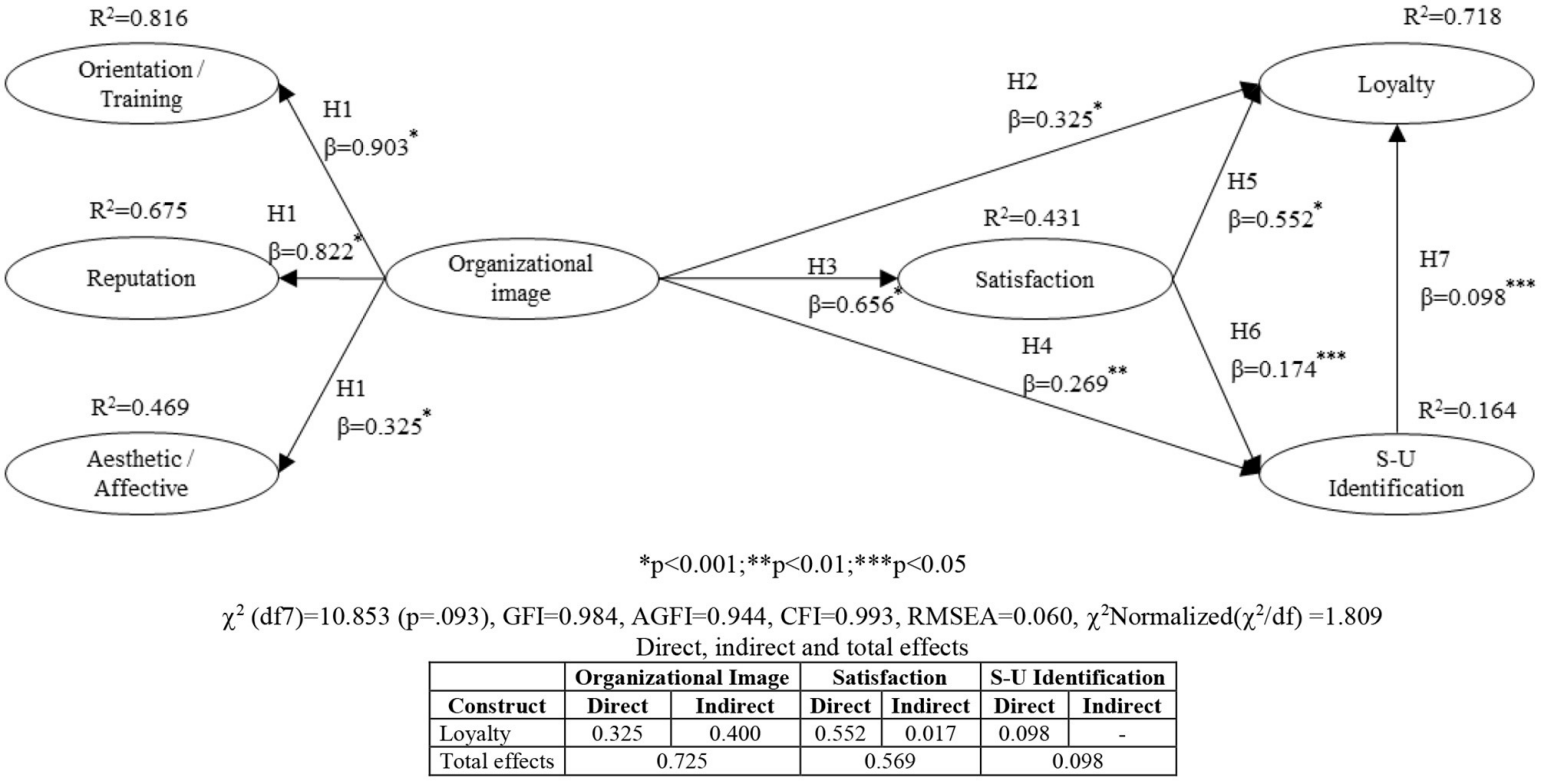
Structural model.

The research hypotheses were tested (Figure 2). The structural model has good adjustment measures; all the indices are higher than the recommended minimum values. The standardized coefficients (β) that show the weights of the direct effects of one variable on another and the direction (hypothesis) are all significant at the *p* < 0.001, 0.01 and 0.05 level. *R*^2^ indicates the amount of variance of the constructs that is explained by the model. The model explains 71.8% of loyalty and 16.4% of the S-U identification construct. All hypotheses are corroborated.

## Discussion

The results of the Structural Model (Figure 2) support the explanatory capacity of the proposed theoretical model. The student loyalty dimension has an *R*^2^ = 0.718; considering the criteria proposed by Hu and Bentler (1995), it is> 0.67 therefore, its explanatory capacity is strong. All the hypotheses proposed are corroborated with significant β (*p*> 0.001, 0.01 and 0.05), β that indicate the relative importance of the dependent variable. The background variables of loyalty and that make up the explanatory capacity of the model are image, satisfaction and S-U identification.

Regarding organizational image, H1 is corroborated, perceived image is a multidimensional construct with cognitive and affective components. In this research, the structure is made up of three dimensions, orientation/training, reputation and aesthetic/affective, the first two with a high explanatory capacity in the model (*R*^2^ = 0.816 and 0.675, respectively) and aesthetic/affective has a good capacity with an R^2^ = 0.469. These results are corroborated in the study by Beerli and Díaz (2003). There are many investigations that corroborate that it is a multidimensional concept, although they do differ in their dimensions. Nguyen and Leblanc (2001) point out two components; functional (tangible characteristics) and emotional (psychological aspects such as feelings and attitudes toward the organization). For Beerli et al. (2002), there are two components, cognitive image (beliefs/knowledge about the organization) and affective image (feelings, emotions and benefits sought). Galiniené et al. (2009) identify three; cognitive image, emotional-affective image, general image and Nolan and Harold (2010) provide two, instrumental attributes and symbolic meanings.

This construct (organizational image) influences directly (β = 0.325, *p* < 0.001) (hypothesis H2) and indirectly (β = 0.400) in building student loyalty through satisfaction (hypothesis H3) with β = 0.656, *p* < 0.001 and SU identification (hypothesis H4) (β = 0.269, *p* < 0.01). The causal relationship proposed by hypothesis H2 (image → loyalty) is also corroborated by the investigations carried out by (Martensen et al., 1999; Nguyen and Leblanc, 2001; Beerli et al., 2002; Beerli and Díaz, 2003; Alves and Raposo, 2010). The relationship between image → satisfaction is corroborated by Martensen et al. (1999), Nguyen and Leblanc (2001), Beerli et al. (2002); Helgesen and Nesset (2007); Alves and Raposo (2010). Finally, the relationship between image → S-U identification is corroborated among others, by Dutton et al. (1994); Bhattacharya et al. (1995); Kreiner and Ashforth (2004); Ahearne et al. (2005); Cornwell and Coote (2005).

Regarding satisfaction, it influences loyalty directly (β = 0.552, *p* < 0.001) (H5) and indirectly and weakly through the S-U identification construct with β = 0.174, *p* < 0.05. These relationships are also corroborated by other empirical investigations such as Alves and Raposo (2004); Gonçalves et al. (2004); Schertzer and Schertzer (2004); Taylor et al. (2004); Tsarenko et al. (2004); Marzo et al. (2005). The explanatory capacity of satisfaction is good with an *R*^2^ = 0.431. Finally, the explanatory capacity of S-U identification is low (*R*^2^ = 0.164) and its influence on loyalty is also low (β = 0.098, *p* < 0.05).

## Conclusions and Implications

The structural model proposed and empirically validated allows us to understand how students' loyalty (attitudinal and behavioral) toward the university center where they study is formed. The results empirically show that the organizational image with a total effect on the model of 0.725 on organizational loyalty is the key variable to influence students' decision to continue taking new studies at the center, as well as to recommend it to other people. Therefore, the greater the positive perception of the organizational image of the university center is, the greater their satisfaction with the center will be, which results in an increase in student loyalty to the center in which they study. However, their identification levels that are made operational through the attitude of being able to defend it and feel part of it are not configured as a relevant variable in the process of increasing loyalty levels.

These results have major implications for university center managers who want to retain their current students and attract potential students. So, in order to increase loyalty, they must focus their efforts on improving the image of the center and students' satisfaction. They must pay special attention to improving the guidance that the educational center has toward its stakeholders, society, company and especially toward its students, as well as improving the training quality it provides and working on it so that it is perceived by the student. They should also focus their efforts on improving its reputation, understood as prestige among its stakeholders. And finally, improve both physical aspects of the university center and emotional aspects related to image.

Finally, this research has two limitations. The first one refers to the fact that the model has been contrasted based on the opinions of the students of a specific university center in Spain. This limits the generalization of the results to a certain extent, which leads to proposing as a future line of research, to extend the study to other university centers, both within Spain and in other countries in order to compare results and contrast the model. The second limitation is related to the cross section of the research, as it was carried out at a specific point in time, so it would be interesting to carry out the study longitudinally to observe possible variations in students' perceptions over time. A third limitation is the use of a structured questionnaire that limits the responses of the respondents to the questions asked. However, we have overcome this limitation by calculating the Common Method Bias.

## Data Availability Statement

The raw data supporting the conclusions of this article will be made available by the authors, without undue reservation.

## Ethics Statement

Ethical review and approval was not required for the study on human participants in accordance with the local legislation and institutional requirements. Written informed consent from the participants was not required to participate in this study in accordance with the national legislation and the institutional requirements.

## Author Contributions

All authors listed have made a substantial, direct and intellectual contribution to the work, and approved it for publication. JÁ-G, MdR-R, CO, and AD-S: conceptualization, investigation, methodology, formal analysis, writing—original draft, preparation, and writing—review and editing. All authors have read and agreed to the published version of the manuscript.

## Conflict of Interest

The authors declare that the research was conducted in the absence of any commercial or financial relationships that could be construed as a potential conflict of interest.

## Publisher's Note

All claims expressed in this article are solely those of the authors and do not necessarily represent those of their affiliated organizations, or those of the publisher, the editors and the reviewers. Any product that may be evaluated in this article, or claim that may be made by its manufacturer, is not guaranteed or endorsed by the publisher.

## Appendix

**APPENDIX:** Measurement scales.

**Organizational Image**
*IM1—It is a Faculty that is closely-related to its students*
IM2—It is a Faculty oriented and concerned by its students
*IM3—A good atmosphere is perceived in the Faculty*
IM4—This Faculty has a good team of teaching staff, highly qualified and up-to-date (Quality of teaching staff)
IM5—It is a Faculty that provides good training
*IM6—It is a faculty that provides practical training*
IM7—This Faculty is recognized for its educational quality
IM8—The complementary courses offered by this Faculty are excellent
IM9—In general terms, teaching at the Faculty is good
IM10—Student information systems are good
IM11—It is a Faculty that is closely-related to society
IM12—It is a Faculty with prestige
IM13—The Faculty has a good reputation
IM14—This faculty has a good infrastructure (building) or facilities
IM15—This faculty has good material resources
IM16—This Faculty has a wide range of degrees
IM17—It is a modern Faculty
IM18—It is an Innovative Faculty
IM19—I perceive a good quality of the services provided in this Faculty
IM20—This Faculty has good administrative management
*IM21—This Faculty shares Social Responsibility activities*
IM22—It is a pleasant Faculty
IM23—It is a stimulating Faculty
IM24—It is a dynamic faculty
IM25—It is a cheerful Faculty
**Satisfaction**
S1-Your decision to have chosen the Faculty was the right one
S2-Your experience at the Faculty has met the expectations you had
S3-In general, you are satisfied with your decision of having chosen the Faculty to study
**S-U Identification**
SU1—When someone criticizes the Faculty you take it as a personal insult
SU2—You are interested in what people think about the Faculty
SU3—When you talk about the Faculty, you normally say “we”
SU4—When the Faculty is successful, you feel as if you have also been successful
SU5—When someone praises the Faculty, you take it as a personal compliment
SU6—It bothers you that some media news criticizes the Faculty
**Loyalty**
L1—If you had to take other courses, conferences or professional improvement studies, you would surely consider the Faculty as your first option
L2—If someone asked you for advice, you would recommend the Faculty
L3—If you had the opportunity, you would discuss positive things about the Faculty with your friends and family
L4—You would encourage family and friends to study at this Faculty

*Deleted items are shown in italics*.
